# Graphitic Carbon Nitride Nanosheets Decorated with Zinc-Cadmium Sulfide for Type-II Heterojunctions for Photocatalytic Hydrogen Production

**DOI:** 10.3390/nano13182609

**Published:** 2023-09-21

**Authors:** Ammar Bin Yousaf, Muhammad Imran, Muhammad Farooq, Samaira Kausar, Samina Yasmeen, Peter Kasak

**Affiliations:** 1 Center for Advanced Materials, Qatar University, Doha P.O. Box 2713, Qatar; 2 Hefei National Laboratory for Physical Sciences at Microscale, University of Science and Technology of China, Hefei 230026, China; imran345@mail.ustc.edu.cn; 3 Interdisciplinary Graduate School of Science and Technology, Shinshu University, Ueda 386-8567, Japan; 4 Department of Chemistry, National Science College, Satellite Town, Gujranwala 52250, Pakistan; samairakasr@gmail.com (S.K.); samina.yasmn@gmail.com (S.Y.)

**Keywords:** Heterojunction, photocatalytic H_2_ production, carbon nitride, nanosheets, ZnCdS

## Abstract

In this study, we fabricated graphitic carbon nitride (*g*-C_3_N_4_) nanosheets with embedded ZnCdS nanoparticles to form a type II heterojunction using a facile synthesis approach, and we used them for photocatalytic H_2_ production. The morphologies, chemical structure, and optical properties of the obtained *g*-C_3_N_4_–ZnCdS samples were characterized by a battery of techniques, such as TEM, XRD, XPS, and UV-Vis DRS. The as-synthesized *g*-C_3_N_4_–ZnCdS photocatalyst exhibited the highest hydrogen production rate of 108.9 μmol·g^−1^·h^−1^ compared to the individual components (*g*-C_3_N_4_: 13.5 μmol·g^−1^·h^−1^, ZnCdS: 45.3 μmol·g^−1^·h^−1^). The improvement of its photocatalytic activity can mainly be attributed to the heterojunction formation and resulting synergistic effect, which provided more channels for charge carrier migration and reduced the recombination of photogenerated electrons and holes. Meanwhile, the *g*-C_3_N_4_–ZnCdS heterojunction catalyst also showed a higher stability over a number of repeated cycles. Our work provides insight into using *g*-C_3_N_4_ and metal sulfide in combination so as to develop low-cost, efficient, visible-light-active hydrogen production photocatalysts.

## 1. Introduction

The demand for energy is rising steadily as the global population grows and living standards improve. Hydrogen is considered a clean, plentiful, and secure energy source for addressing this need [1]. The tremendous energy output of hydrogen combustion, which is far higher than that of gasoline or any other fossil fuel, makes it a better and more efficient alternate fuel. As no toxic byproducts are produced during hydrogen combustion, it is also considered ecologically safe [2]. However, carbon dioxide is usually produced during the steam reforming of hydrocarbons and coal for hydrogen production. To avoid producing greenhouse gases, finding workable alternatives is essential. One viable solution to the present energy and environmental dilemma is using solar energy to produce hydrogen from water on the surface of a catalyst [3,4]. Since their first use on the surface of TiO_2,_ semiconductor photocatalysts have been widely utilized for the photolysis of water [5]. To maximize the use of solar power, various attempts have been made to find renewable, efficient photocatalysts with an excellent visible-light response [6,7].

Carbon nitride (*g*-C_3_N_4_) graphitic material has been used as a C-related and potential candidate, with the characteristics of a metal-free photocatalyst in terms of hydrogen evolution and organic degradation due to its suitable band gap (ca. 2.7 eV) [8]. Moreover, graphitic carbon-nitride materials with a similar chemistry to that of graphene (though more exceptional with regards to specific chemical characteristics (i.e., electronic and optical properties) compared to those of previously studied 2D-materials) are a prestigious choice for energy applications. Additionally, the electronic structure of the triazine units in g-C_3_N_4_ forms conjugated graphitic planes, which are very stable and responsive to visible light. However, its photocatalytic performance is severely impacted by both the negligible absorption or lack of absorption under the visible portion of light irradiation (beyond 460 nm) and the fast recombination for photo-induced charge carriers species [9]. To enhance the catalytic performance and promote the separation of photo-generated holes and electrons, a number of strategies have been adopted, such as developing heterojunctions, and another semiconductor is usually coupled with *g*-C_3_N_4_, such as *g*-C_3_N_4_/CdS, *g*-C_3_N_4_/TiO_2_, *g*-C_3_N_4_/MoO_3_, *g*-C_3_N_4_/BiVO_4_, and *g*-C_3_N_4_/InVO_4_ [10,11,12,13,14]. However, the complex preparation process and catalyst deterioration over a few cycles make it harder to use it on a broader industrial scale.

Among other alternatives, solid sulfide solutions, such as ZnIn_2_S_4_, CdIn_2_S_4_, ZnCdS, and Mn_x_Cd_1-x_S, have been used in photocatalytic hydrogen production because of their appropriate band gap, high visible-light response, and tunable structure [15,16,17,18]. The easily tunable band structure and superior reducing ability of ZnCdS mean that it stands out among solid sulfide solutions [19]. However, it does have several drawbacks, including an inadequate photo-generated carrier transmission efficiency, low solar energy consumption, and rapid electron-hole pair recombination, which severely restricts its photocatalytic efficacy [20,21]. The charge recombination efficiency and energy output can be improved by combining ZnCdS with another photocatalyst, offering more active sites and reaction sites to promote oxidation and reduction processes.

Herein, we demonstrated a simple strategy to fabricate *g*-C_3_N_4_ with ZnCdS to form a type II heterojunction. The conjugated graphitic planes of *g*-C_3_N_4_ nanosheets provided a large surface area for ZnCdS, which acted efficiently so as to use the charge carrier and enhance H_2_ production.

## 2. Materials and Methods

### 2.1. Chemicals

Melamine (C_3_H_6_N_6_), sodium hydroxide (NaOH), zinc acetate [Zn(OAc)_2_], cadmium acetate [Cd(OAc)_2_], sodium sulfide nonahydrate [Na_2_S·9H_2_O], and sodium sulfite (Na_2_SO_3_) of analytical-grade purity were purchased from Sigma-Aldrich and used as received without further purification.

### 2.2. Characterization

The nanosized structure and morphology of the as-synthesized samples were determined by transmission electron microscope (TEM, JEM-2100F, JEOL, Tokio, Japan), an accelerating voltage of 200 kV was used, and the sample was deposited on microscopical grids for analysis. The diffraction characteristics, such as the crystal phase of the samples, was analyzed by powder X-ray diffraction (XRD) measurements with a Philips X’Pert Pro Super diffractometer with Cu-Kα radiation (λ = 1.54178 Å) at an operating voltage of 40 kV and current of 200 mA. The elemental composition of the prepared catalyst was determined using X-ray photoelectron spectroscopy (XPS) analysis (the measurements were carried out using SPeCS system (PHOIBOS 150, Berlin, Germany) with Al Kα radiation (hʋ¼ 1486.6 eV). The spectra were acquired in the constant analyzer energy mode with a pass energy of 100 eV, 10 kV, and 10 mA emission current for the survey. The individual high-resolution scans were carried out with a pass energy of 10 eV, 15 kV, and 15 mA emission current. Spectral calibration was determined using the automated calibration routine and the internal C 1s standard. The surface compositions (in atomic %) were determined by considering the integrated peak areas of detected atoms and the respective sensitivity factors. The UV−vis diffuse reflectance spectra (DRS) for the band-gap calculation and absorption behavior of the samples were recorded with a Shimadzu spectrophotometer (2501 PC model) in the 200 to 800 nm region. Photoelectrochemical tests were performed with a CHI-660B potentiostat (Chenhua Instrument Co., Shanghai, China) with a three-electrode setup (modified Ti foil as the working electrode, Ag/AgCl as the reference electrode, and Pt wire as the counter electrode) in 0.1 M Na_2_SO_4_ solution. The Fourier transform infrared (FTIR) spectra were performed on a spectrometer (Nicolet iS50, Thermo Scientific, Waltham, MA, USA) to attain the spectra in the middle infrared region (4000–600 cm^–1^), applying a ZnSe crystal permitting ~1.7 µm penetration depth and using an average of 64 scans with a resolution of 1 cm^–1^. Suitable contact between the analyzed samples and the FTIR crystal was guaranteed by means of a pressure clamp, permitting one to attain a high spectral quality. The electrochemical impedance spectroscopy (EIS) was recorded with −0.6 V bias, and the frequency ranged from 1 Hz to 100 kHz with an alternating current signal amplitude of 5 mV.

### 2.3. Synthesis of g-C_3_N_4_ Nanosheets

The preparation of *g*-C_3_N_4_ was performed in an alumina crucible with a cover, which could form a semi-closed atmosphere to prevent the sublimation of precursors. Melamine powder (3 g) was placed into the crucible and heated to a temperature of 530 °C in a tube furnace with N_2_ atmosphere. Then, the sample was naturally cooled to room temperature, collected, and stored for further use.

### 2.4. Synthesis of g-C_3_N_4_–ZnCdS Heterojunction

The fabrication of *g*-C_3_N_4_ nanosheets with ZnCdS was achieved following our previous protocol, described previously in reference [22]: first, *g*-C_3_N_4_ nanosheets were dispersed in 100 mL of DI water, and then the proper quantity of cadmium acetate and zinc acetate was slowly poured into the dispersion to achieve 10wt% of ZnCdS on the *g*-C_3_N_4_ nanosheets. The mixture’s pH was adjusted to 7.0 with dilute aqueous sodium hydroxide. Subsequently, aqueous Na_2_S solution was added dropwise. The samples were stirred at room temperature for 12 h and extracted using centrifugation, washed with ethanol and water, and then dried overnight at 60 °C in a vacuum oven. Finally, the obtained powders were calcined for 2 h at 400 °C under nitrogen flow with a heating rate of 5 °C/min.

### 2.5. Photo and Electrochemical Measurements

Measurement for photocatalytic hydrogen production was performed under visible light over as-synthesized samples by means of a vacuum-closed cell circulation system and catalyst powder [22]. A 300 W Xe lamp was used as a light source with a filter (λ ≥ 420 nm) to block UV light. The experiment was performed by dispersing 100 mg of catalyst in 100 mL aqueous solution containing 0.1 M Na_2_S and 0.1 M Na_2_SO_3_ as sacrificial agents. Gas chromatography (Agilent (Santa Clara, CA, USA), 6820, TCD detector, N_2_ carrier) was used to determine the amount of H_2_ produced.

Electrochemical and photoelectrochemical measurements were carried out in a setup of three electrode quartz cells and 0.1 M Na_2_SO_4_ solution with Ag/AgCl and Pt wire as a reference and counter electrode, respectively, while the catalyst film on Ti foil was used as a working electrode. The catalyst films for electrochemical measurements were prepared by applying an appropriate amount of catalyst suspension onto Ti foil. For the measurements, the electrodes were pressed against the round shape of an electrochemical cell with a working area of 4.0 cm^2^. Photo-electrochemical test systems were composed of a CHI 660B electrochemistry potentiostat (Shanghai Chenhua Limited, Shanghai, China). The amperometric photocurrents were observed by an on/off switch with a bias voltage of 0.5 V under visible light. The electrochemical impedance spectroscopy (EIS) was recorded with −0.6 V bias, and the frequency ranged from 1 Hz to 100 kHz with an alternating current signal amplitude of 5 mV.

## 3. Results

**Figure 1:**
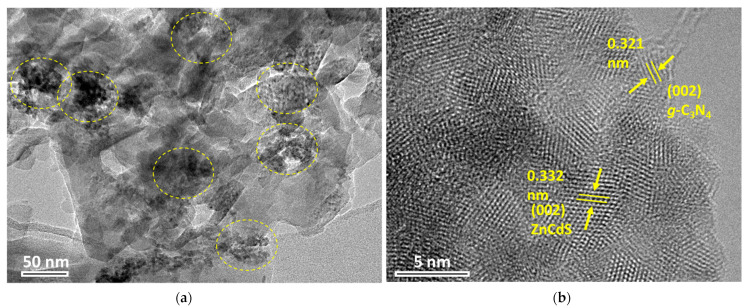
Transmission electron microscopy (TEM) images for as-prepared *g*-C_3_N_4_–ZnCdS samples: (**a**) lower magnifications and (**b**) higher magnifications.

The morphologies of the as-prepared samples were determined by transmission electron microscope (TEM). As can be seen in Figure 1 and Figure S1, *g*-C_3_N_4_ exhibited a planar nanosheet structure. ZnCdSs were small, irregularly shaped nanoparticles distributed onto the nanosheets (yellow circles in Figure 1a). The high-resolution TEM image shows clear lattice dispersing of *g*-C_3_N_4_ and ZnCdS, with values of 0.321 nm and 0.332 nm corresponding to a (002) plane distance (Figure 1b).

**Figure 2:**
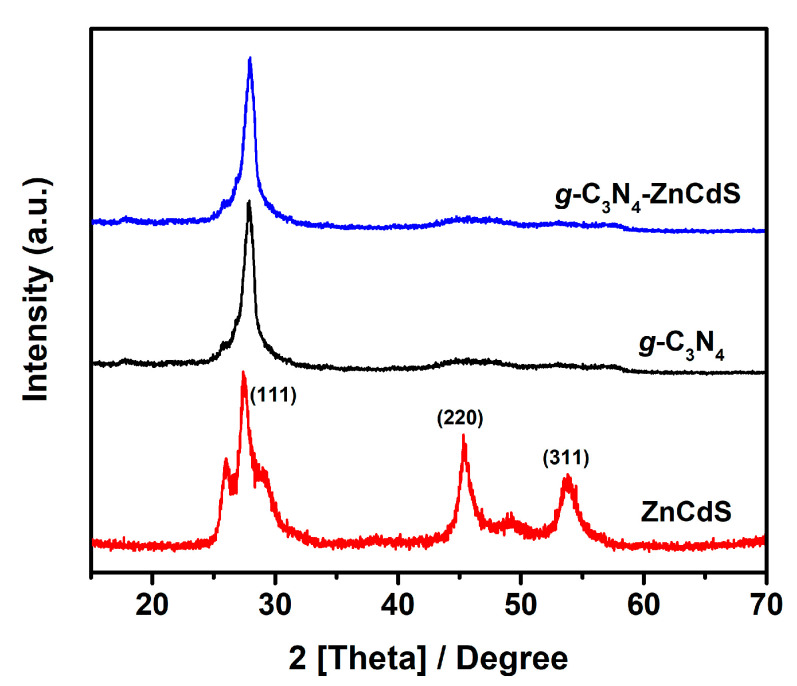
XRD spectrum of *g*-C_3_N_4_ (black line), ZnCdS (red line), and *g*-C_3_N_4_–ZnCdS (blue line) samples.

The XRD results of *g*-C_3_N_4_, ZnCdS, and *g*-C_3_N_4_–ZnCdS heterojunctions are depicted in Figure 2. As shown in the case of pristine *g*-C_3_N_4_, the peak at 27.9° for the (002) diffraction plane was derived from interplanar stacking peaks of conjugated aromatic systems of C_3_N_4_. The peak was well matched with JCPDS #87-1526 of *g*-C_3_N_4_ [23]. The XRD results of ZnCdS showed peaks indexed at 27.34°, 45.32°, and 53.66° corresponding to the (111), (220), and (311) planes of the cubic phase of the Zinc blend related to ICSD #80-0020. In the *g*-C_3_N_4_–ZnCdS heterojunction, there were no clear diffraction peaks of ZnCdS because of its relatively low levels and smaller size compared to *g*-C_3_N_4_ [22]. The lower amount of ZnCdS was also confirmed by XPS and ICP analysis, which showed values of 7.3wt% and 8.4wt%, respectively, close to the experimentally adjusted value of 10wt%.

Fourier transform infrared spectroscopy (FTIR) was further conducted to specifically verify the existence of characteristic functional groups related to g-C_3_N_4_ and those associated with the coupled second part ZnCdS, and their proportions. Figure S2 shows the Fourier transform infrared (FTIR) spectra of *g*-C_3_N_4_ and *g*-C_3_N_4_-ZnCdS samples. The g-C_3_N_4_ spectrum shows that the broad band at 3161 cm^−1^ is mainly attributed to the N-H stretching vibration of *g*-C_3_N_4_. The peaks at 1242, 1320, 1408, 1459, 1572 and 1636 cm^−1^ can be ascribed to the stretching vibration modes of C–N heterocycles and that the peak at 809 cm^−1^ is related to the vibration modes of the s-triazine rings. These distinct peaks further confirm the successful preparation of the *g*-C_3_N_4_ structure [24,25]. The FTIR spectra of the g-C_3_N_4_-ZnCdS heterostructure exhibit the main characteristic peaks of pure *g*-C_3_N_4_. This result verified that the g-C_3_N_4_ structure was well retained after the heterostructure’s formation.

**Figure 3:**
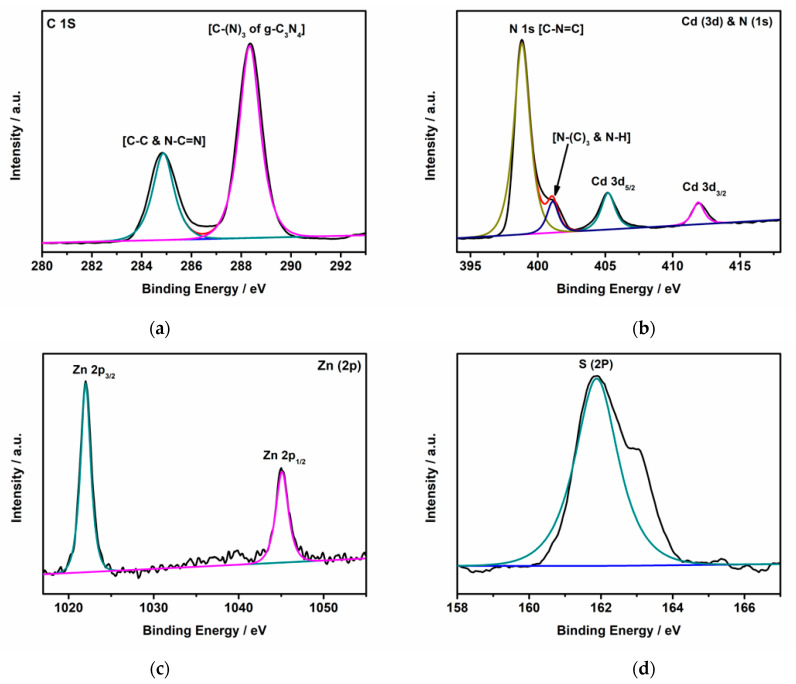
High-resolution XPS spectrum for a *g*-C_3_N_4_–ZnCdS sample. Scans for (**a**) C1s, (**b**) Cd3d and N1s, (**c**) Zn2p, and (**d**) S2p regions.

X-ray photoelectron spectroscopic (XPS) analysis was applied to determine the elemental composition of the prepared catalyst and chemical state of particular elements. The survey spectrum of the *g*-C_3_N_4_–ZnCdS heterojunctions, shown in Figure S3, indicates that the sample primarily comprised C, N, Zn, Cd, and S elements. For a further illustration of the elemental signals, high-resolution XPS spectra are provided in Figure 3. In the high-resolution XPS spectra of C1s shown in Figure 3a, the peak positioned at 284.8 eV can be related to sp^2^ carbon atoms (C-C and N-C=N bonding) originating from the surface exotic C in the instrument. The second peak located at 288.3 eV can be attributed to sp^2^ hybridized C bonded to nitrogen [C-(N)_3_ of *g*-C_3_N_4_]. The high-resolution XPS spectrum of N1s shows a large peak centered at 398.8 eV, which can be ascribed to a nitrogen atom bonded to carbon [C-N=C], while the shoulder peak at 401.1 eV can readily be ascribed to N-(C)_3_ and N-H [26,27] (Figure 3b). The high-resolution XPS spectrum of Cd3d shows two spin-orbit components centered at 405.2 eV and 411.9 eV, which correspond to Cd3d_5/2_ and Cd3d_3/2_, respectively (Figure 3b). Similarly, the Zn2p region also shows components indexed at 1022 eV and 1045 eV, ascribed to Zn2p_3/2_ and Zn2p_1/2_, respectively (Figure 3c). Figure 3d denotes the high-resolution spectra of S2p, which exhibit a peak centered at 161.87 eV, which is attributed to the S^2−^ valent state of S in the ZnCdS segment. All the peaks are well-matched with the values reported previously for ZnCdS [28].

**Figure 4:**
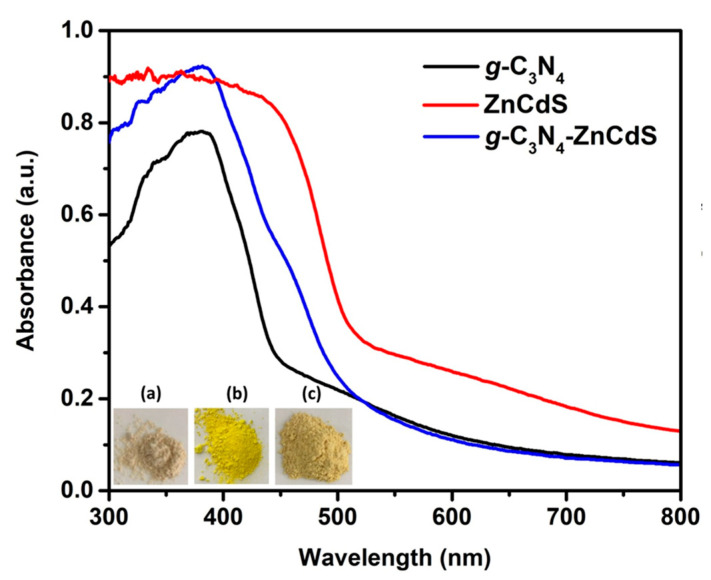
UV–visible diffuse reflectance spectra (DRS), and the insets show images of the (**a**) *g*-C_3_N_4_, (**b**) ZnCdS, and (**c**) *g*-C_3_N_4_–ZnCdS samples.

The optical properties of pure *g*-C_3_N_4_ nanosheets, ZnCdS nanoparticles, and *g*-C_3_N_4_–ZnCdS heterojunctions were measured with UV-vis DRS. As shown in Figure 4, the characteristic absorption peak of pure *g*-C_3_N_4_ nanosheets was about 400 nm, arising from the intrinsic band gap of *g*-C_3_N_4_ at about 2.7 eV, which itself has low visible-light absorption characteristics. On the other hand, ZnCdS showed strong absorption towards the visible region, and the absorption edge extended towards 500 nm. After introducing ZnCdS nanoparticles into g-C_3_N_4_ nanosheets, heterojunction formation showed an increased absorption intensity compared to bare *g*-C_3_N_4_ nanosheets, and the absorption edge also moved towards the visible region. Figures S4 and S5 display the related *Tauc* plots for the evaluation of band-gap energies. Band-gap energies were obtained from analysis of the plot and the intercept of the tangent of the curve (αhν)^2^ and (αhν)^1/2^ vs. (hν), as previously reported [29].The value of the exponent in αhν (2 and ½) depends on the transition in a semiconductor: 1/2 for a direct transition and 2 for an indirect transition. The indirect band gap of *g*-C_3_N_4_ and the *g*-C_3_N_4_-ZnCdS heterostructure was determined by plotting the value of (*αhν*)^1/2^ vs. *hν* (Figure S4). On the other hand, ZnCdS has a direct transition; hence, the band gap of ZnCdS was calculated by plotting the value of (*αhν*)^2^ vs. *hν* (Figure S5). The calculated band-gap energies for *g*-C_3_N_4_ nanosheet, ZnCdS nanoparticle, and *g*-C_3_N_4_–ZnCdS heterojunction samples were determined from the *Tauc* plot as being 2.67 eV, 2.25 eV, and 2.46 eV, respectively. After the introduction of ZnCdS nanoparticles, the large band gap of *g*-C_3_N_4_ nanosheets decreased, which supports their photocatalytic performance.

**Figure 5:**
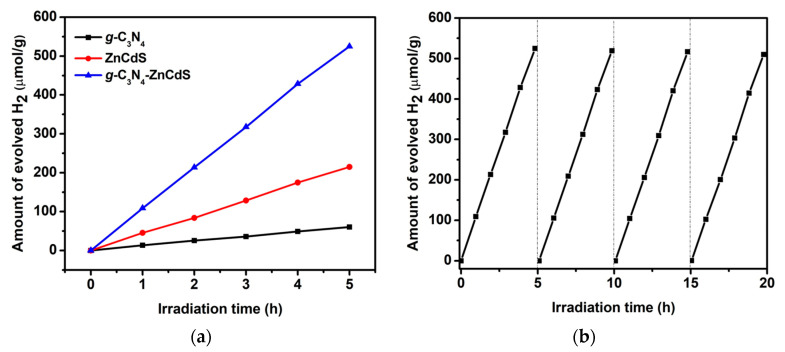
(**a**) Photocatalytic H_2_ evolution activities from water splitting on samples from *g*-C_3_N_4_ nanosheets, ZnCdS nanoparticles, and *g*-C_3_N_4_–ZnCdS under visible-light illumination (λ  ≥  420 nm) over 5 h. (**b**) Cyclic tests for photocatalytic H_2_ evolution activities from water solution on *g*-C_3_N_4_–ZnCdS for four consecutive cycles.

The photocatalytic hydrogen evolution ability of bare *g*-C_3_N_4_ nanosheets, ZnCdS nanoparticles, and *g*-C_3_N_4_–ZnCdS heterojunctions was evaluated under visible-light irradiation, as shown in Figure 5a. The H_2_ production rate for*g*-C_3_N_4_ was observed at 13.5 μmol·g^−1^·h^−1^. In comparison, ZnCdS was 45.3 μmol·g^−1^·h^−1^. Compared to bare samples, the *g*-C_3_N_4_–ZnCdS heterojunction showed an increase in photocatalytic H_2_ production (108.9 μmol·g^−1^·h^−1^). This was about eight times higher than *g*-C_3_N_4_ and 2.4 times higher than ZnCdS. The increase in the H_2_ production rate indicated that a heterojunction formed between the individual components, which facilitated the mobility of the charge carrier and enhanced the photocatalytic performance. Another issue to consider in the applicability of photocatalysts is their performance in reusability. Therefore, reusability experiments were performed for the *g*-C_3_N_4_–ZnCdS heterojunctions, and after each run, the catalyst was recovered by centrifugation, washed with water and ethanol, and reused. As displayed in Figure 5b, the hydrogen generation rate was remarkably stable over five cycles (94% retention rate with a value decrease from 108.9 to 102.3 μmol·g^−1^·h^−1^), indicating the excellent stability and sustainable utilization of the photocatalyst.

**Figure 6:**
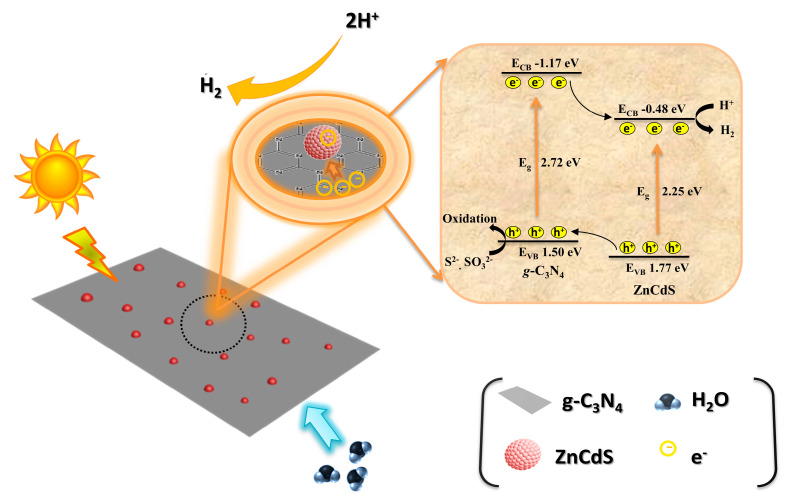
A schematic representation showing the photocatalytic process, band positions, and charge transfer process for g-C_3_N_4_–ZnCdS catalyst.

In order to explore how the *g*-C_3_N_4_–ZnCdS heterojunction shows a better photocatalytic performance compared to individual components, the electron transfer mechanism was revealed, as shown in Figure 6. The conduction and valence band potentials of photocatalyst *g*-C_3_N_4_ nanosheets and ZnCdS nanoparticles were determined by a Mott−Schottky plot to gain insight into the photocatalysis mechanism (Figures S6 and S7). The conduction band (CB) edge potentials of *g*-C_3_N_4_ nanosheets and ZnCdS nanoparticles were determined to be −1.17 V and −0.48 V, respectively. Based on the band-gap positions, the valence band (VB) edge potentials of *g*-C_3_N_4_ nanosheets and ZnCdS nanoparticles were calculated to be 1.50 V and 1.77 V, respectively. The corresponding band structure diagram can thus be schemed and is shown in Figure 6. Both the valence and conduction bands for ZnCdS were lower compared to *g*-C_3_N_4_, which facilitated the formation of type II heterojunctions. Upon visible-light irradiation, both ZnCdS and *g*-C_3_N_4_ can be excited, and then electrons from the CB of *g*-C_3_N_4_ can be transferred into the CB of ZnCdS, then reacting with H^+^ for H_2_ production. At the same time, the photo-induced holes of *g*-C_3_N_4_ and ZnCdS can be used by oxidizing agents [30,31].

**Figure 7:**
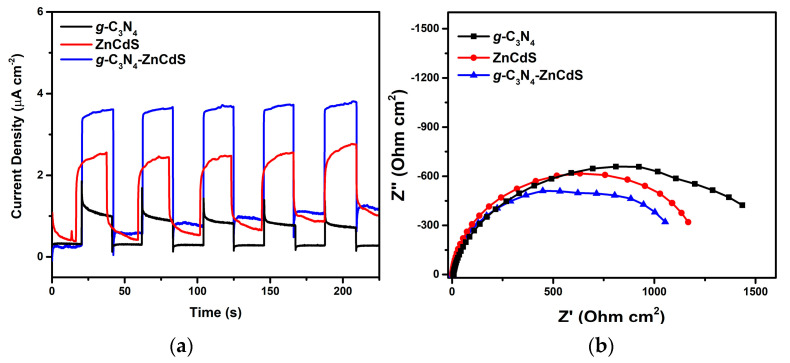
(**a**) Cyclic performance of the response of the current from as-synthetized photocatalysts vs. time for *g*-C_3_N_4_, ZnCdS, and *g*-C_3_N_4_–ZnCdS (irradiated at λ ≥ 420 nm). (**b**) Nyquist plots from EIS measurement of *g*-C_3_N_4_, ZnCdS, and *g*-C_3_N_4_–ZnCdS.

The photocatalytic conjecture was further verified by transient photocurrent response. It was recorded for *g*-C_3_N_4_, ZnCdS, and *g*-C_3_N_4_–ZnCdS heterojunctions. Figure 7a shows *I-t* curves for as-synthesized electrode films with five ON-OFF intermittent visible-light irradiation consecutive cycles [32]. The responses of the photocurrent appeared in all the electrodes instantly as the light was turned on and then rapidly declined to (nearly) zero as the light was off, which was reproducible and stable in five consecutive cycles. Under similar conditions as for irradiation, the photo-current value of the ZnCdS electrode was about twice that of bare *g*-C_3_N_4_, suggesting that there was a low recombination and fast migration of photogenerated electrons on the *g*-C_3_N_4_ nanosheets. Additionally, after heterojunction formation between individual components, *g*-C_3_N_4_–ZnCdS showed a much higher photocurrent value by about 2.6 times, confirming that the photogenerated electrons from the *g*-C_3_N_4_ were taking part in the electron transfer process and shifted to the CB of ZnCdS efficiently.

EIS is another efficient technique to observe the charge transfer efficiency and the interface reaction ability, which explains charge transfer resistance [33]. Figure 7b shows the Nyquist plots of *g*-C_3_N_4_, ZnCdS, and *g*-C_3_N_4_–ZnCdS heterojunctions. The smaller diameter implied a low impedance and fast interface charge transfer. The *g*-C_3_N_4_–ZnCdS heterojunction had the smallest diameter compared to bare samples, and it also showed less charge transfer resistance and coincided well with photocurrent response results. Overall, our results showed that the heterojunction formation between *g*-C_3_N_4_ and ZnCdS enabled less recombination and faster photogenerated electron migration, resulting in a higher photocatalytic performance and enhanced durability.

## 4. Conclusions

In summary, we successfully synthesized *g*-C_3_N_4_–ZnCdS heterojunctions via a facile physical mixture and calcination method. The as-synthesized material was characterized using a battery of techniques, such as TEM, XRD, XPS, and UV−vis DRS. The catalysts were used for photocatalytic H_2_ production, and among all synthesized materials, *g*-C_3_N_4_–ZnCdS revealed an enhanced UV−vis-induced photocatalytic performance. Hydrogen production for the *g*-C_3_N_4_–ZnCdS sample was 108.9 μmol·g^−1^·h^−1^ under visible light, which was significantly higher compared to individual components. The photocatalysts also possessed excellent repeatability over five consecutive cycles, with a mere 6% decrease in photocatalytic activity. The higher and modified photocatalytic performance mainly depended on heterojunction formation among the components and heterojunction formation. The transient photocurrent responses and EIS further supported the enhanced performance due to decreased electron-hole recombination and low charge transfer resistance. The facile synthetic approach and better performance of *g*-C_3_N_4_–ZnCdS provides new opportunities for the further study of the photocatalytic process of coupled semiconductors for hydrogen production.

## Data Availability

The data presented in this study are available on request from the corresponding authors.

## Supplementary Materials

The following supporting information can be downloaded at: https://www.mdpi.com/article/10.3390/nano13182609/s1, Figure S1: Low-resolution TEM image of the *g*-C_3_N_4_–ZnCdS catalyst; Figure S2. Fourier transform infrared (FTIR) spectra of *g*-C_3_N_4_ and *g*-C_3_N_4_–ZnCdS samples. Figure S3: XPS full scan survey for the *g*-C_3_N_4_–ZnCdS catalyst. Figure S4: *Tauc* plot for *g-*C_3_N_4_ and *g*-C_3_N_4_-ZnCdS heterostructure. Figure S5: *Tauc* plot for ZnCdS nanoparticles. Figure S6: Mott−Schottky plot collected for *g*-C_3_N_4_. Figure S7: Mott−Schottky plot collected for ZnCdS nanoparticles.
