# Supplementary material for: Graphitic Carbon Nitride Nanosheets Decorated with Zinc-Cadmium Sulfide for Type-II Heterojunctions for Photocatalytic Hydrogen Production
Source: Nanomaterials (Basel). 2023 Sep 21;13(18):2609. doi: 10.3390/nano13182609 (PMC10535485; doi:10.3390/nano13182609)
Supplement: Supplementary file 1 [file nanomaterials-13-02609-s001.zip › nanomaterials-2552553-supplementary.pdf]

Electronic Supporting Information

Graphitic Carbon Nitride Nanosheets Decorated Zinc-Cadmium Sulfide Type-II Heterojunctions for Photocatalytic Hydrogen Production

Ammar Bin Yousaf,[1]† Muhammad Imran,[2]† Muhammad Farooq,[3,]* Samaira Kausar,[4] Samina Yasmeen,[4] and Peter Kasak,[1,]*

[1] Center for Advanced Materials, Qatar University, Doha 2713, Qatar

[2] Hefei National Laboratory for Physical Sciences at Microscale, University of Science and Technology of China, Hefei, Anhui 230026, PR China

[3] Interdisciplinary Graduate School of Science and Technology,, Shinshu University, Ueda 386-8567, Japan

[4] Department of Chemistry, National Science College, Satellite Town, Gujranwala 52250, Pakistan

† : These two authors contributed equally.

*Corresponding Authors E-mails: mufaroouk@gmail.com, 20hs109g@shinshu-u.ac.jp (Muhammad Farooq), peter.kasak@qu.edu.qa (Peter Kasak)

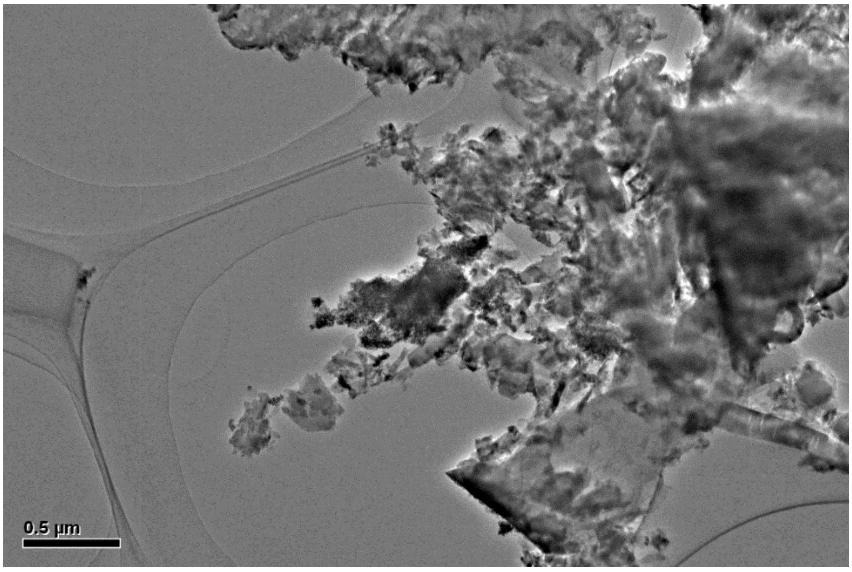

Figure S1: Low resolution TEM image of *g*-C_3N_4–ZnCdS catalyst.

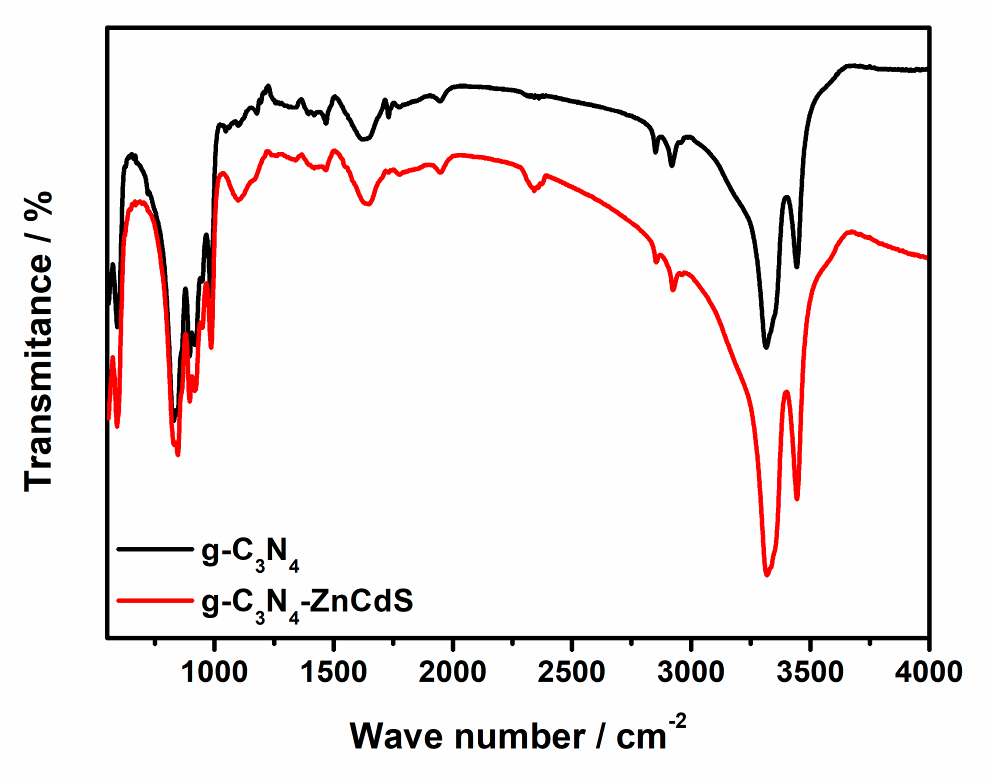

Figure S2. Fourier transform infrared (FTIR) spectra of*g*-C_3N_4 (black line) and *g*-C_3N_4–ZnCdS (blue line) samples.

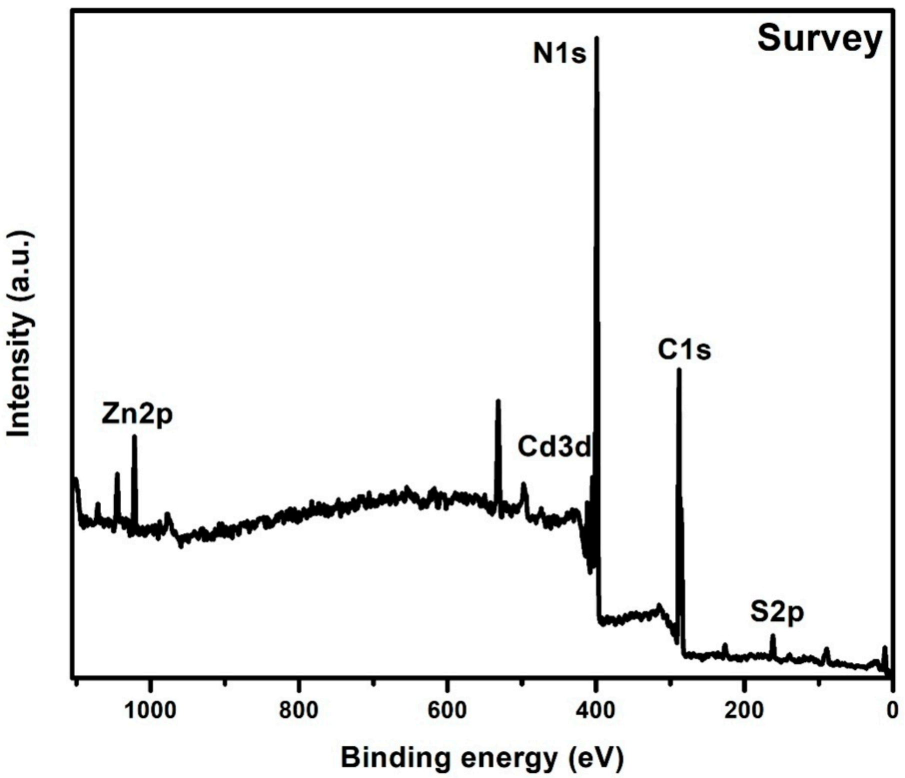

Figure S3: XPS full scan survey for *g*-C_3N_4–ZnCdScatalyst.

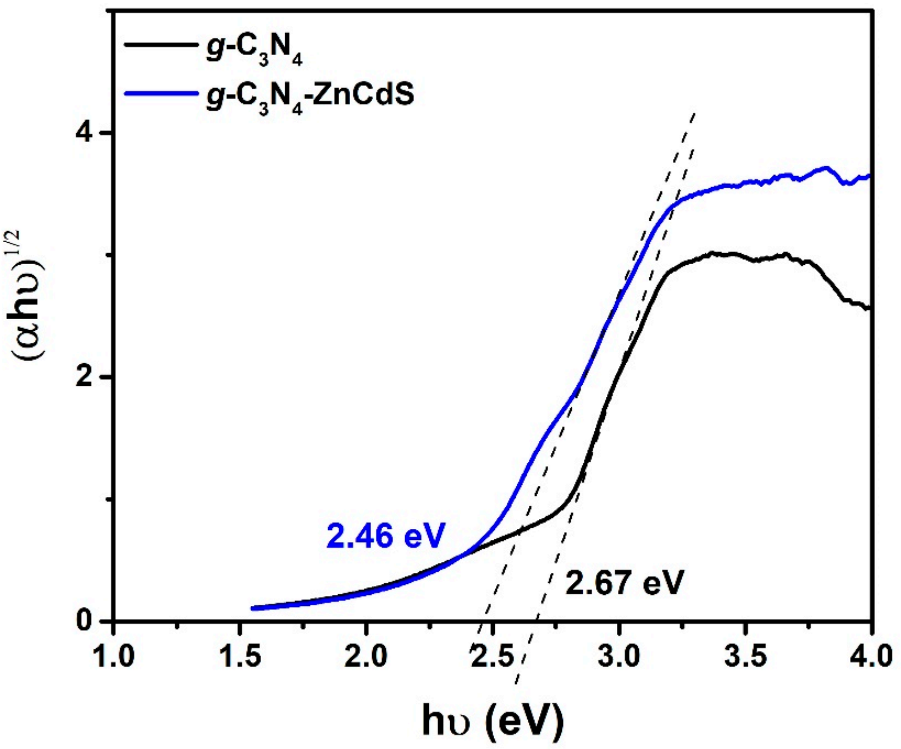

Figure S4: *Tauc* plot for g-C3N4 and g-C3N4-ZnCdS heterostructure.

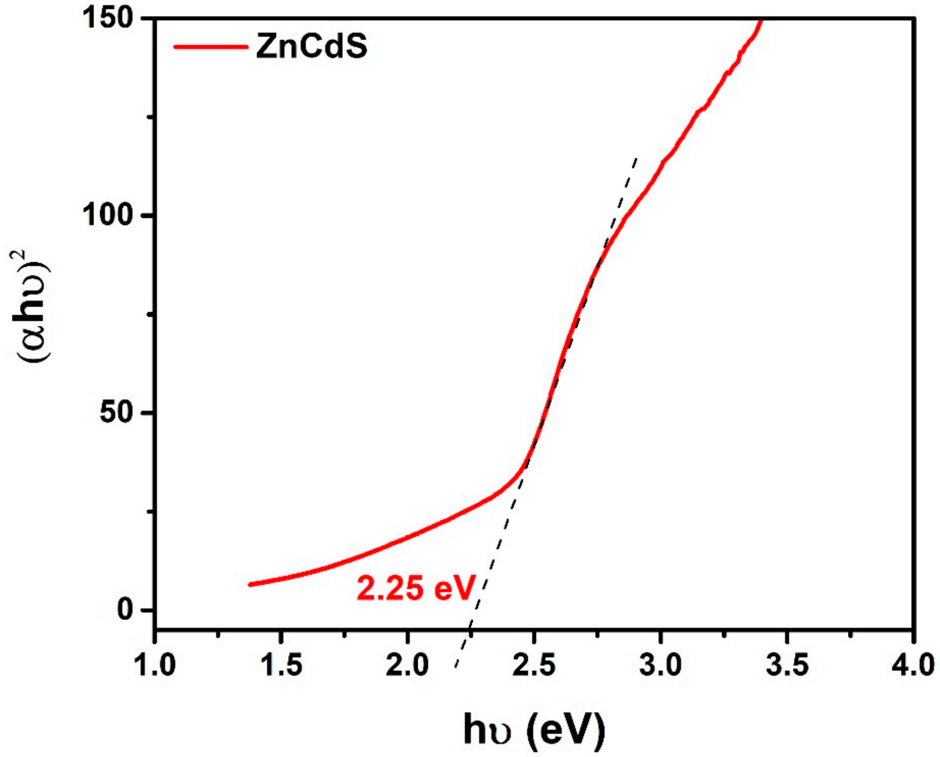

Figure S5: *Tauc* plot for ZnCdS nanoparticles.

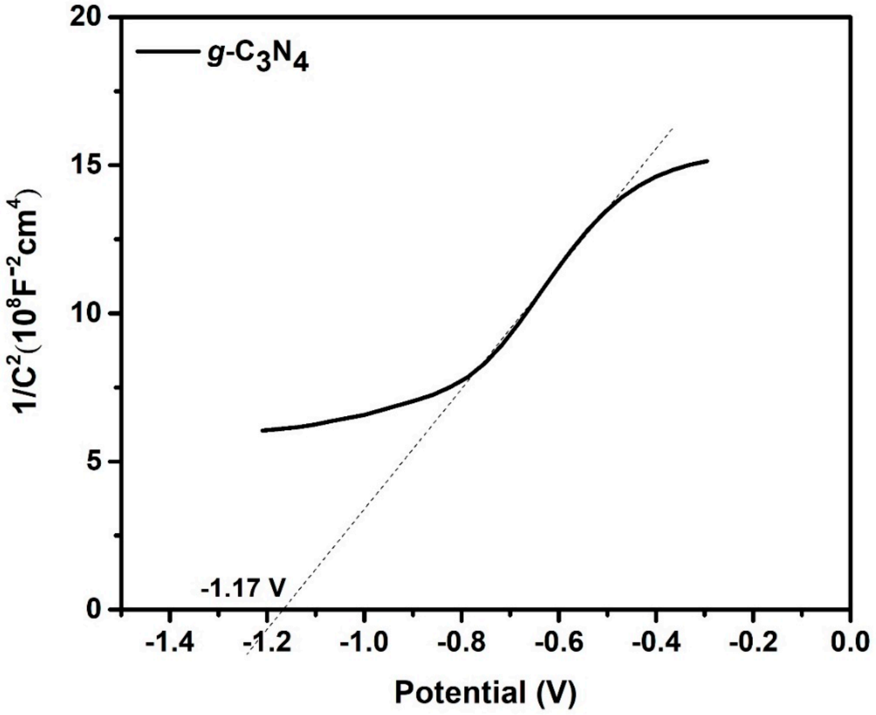

Figure S6: Mott-Schottky plot collected for *g*-C_3N_4.

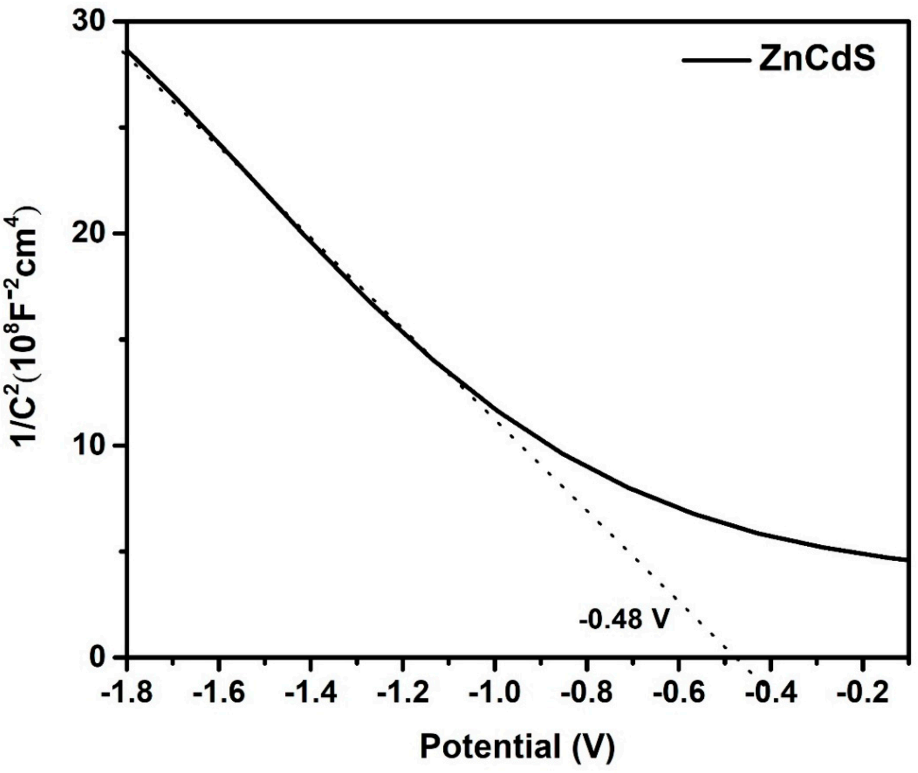

Figure S7: Mott-Schottky plot collected for ZnCdS nanoparticles.
